# Prognosis models for severe and critical COVID-19 based on the Charlson and Elixhauser comorbidity indices

**DOI:** 10.7150/ijms.50007

**Published:** 2020-08-25

**Authors:** Wei Zhou, Xiaoyi Qin, Xiang Hu, Yingru Lu, Jingye Pan

**Affiliations:** 1 Department of Intensive Care Unit, The First Affiliated Hospital of Wenzhou Medical University, Wenzhou, Zhejiang, China.; 2 Department of Hematology, The First Affiliated Hospital of Wenzhou Medical University, Wenzhou, Zhejiang, China.; 3 Department of Endocrine and Metabolic Diseases, The First Affiliated Hospital of Wenzhou Medical University, Wenzhou, Zhejiang, China.

**Keywords:** Charlson comorbidity index, comorbidity, Corona Virus Disease, Elixhauser comorbidity index, length of stay, outcome

## Abstract

**Background**: Corona Virus Disease 2019 (COVID-19) has become a global pandemic. This study established prognostic scoring models based on comorbidities and other clinical information for severe and critical patients with COVID-19.

**Material and Methods**: We retrospectively collected data from 51 patients diagnosed as severe or critical COVID-19 who were admitted between January 29, 2020, and February 18, 2020. The Charlson (CCI), Elixhauser (ECI), and age- and smoking-adjusted Charlson (ASCCI) and Elixhauser (ASECI) comorbidity indices were used to evaluate the patient outcomes.

**Results**: The mean hospital length of stay (LOS) of the COVID-19 patients was 22.82 ± 12.32 days; 19 patients (37.3%) were hospitalized for more than 24 days. Multivariate analysis identified older age (OR 1.064, *P* = 0.018, 95%CI 1.011-1.121) and smoking (OR 3.696, *P* = 0.080, 95%CI 0.856-15.955) as positive predictors of a long LOS. There were significant trends for increasing hospital LOS with increasing CCI, ASCCI, and ASECI scores (OR 57.500, *P* = 0.001, 95%CI 5.687-581.399; OR 71.500, *P* = 0.001, 95%CI 5.689-898.642; and OR 19.556, *P* = 0.001, 95%CI 3.315-115.372, respectively). The result was similar for the outcome of critical illness (OR 21.333, *P* = 0.001, 95%CI 3.565-127.672; OR 13.000, *P* = 0.009, 95%CI 1.921-87.990; OR 11.333, *P* = 0.008, 95%CI 1.859-69.080, respectively).

**Conclusions**: This study established prognostic scoring models based on comorbidities and clinical information, which may help with the graded management of patients according to prognosis score and remind physicians to pay more attention to patients with high scores.

## Introduction

In December 2019, several cases of unexplained pneumonia with a history of exposure to a South China seafood market were seen in hospitals in Wuhan, Hubei Province, China. The pathogen was quickly identified as a novel coronavirus 1 and the World Health Organization (WHO) officially named the disease Corona Virus Disease 2019 (COVID-19). The novel coronavirus, which is similar to severe acute respiratory syndrome coronavirus (SARS-CoV), was designated SARS-CoV-2 by the International Committee on Taxonomy of Viruses 2, 3. On March 11, 2020, the WHO announced that COVID-19 had become a global pandemic. More than 200 countries/territories have reported laboratory-confirmed COVID-19 cases 4. As of April 7, 2020, there have been 1,279,722 confirmed cases and 72,614 deaths globally 4.

Many clinical characteristics and outcomes of COVID-19 have been reported 5-7. Although most patients have mild symptoms and favorable prognoses, older age is associated with poor prognosis for COVID-19 7, 8. Moreover, in a systematic review, Vardavas *et al*. showed that smoking was most likely to be associated with negative progression and adverse outcomes of COVID-19 9. In addition to older age and smoking, the COVID-19 patients with adverse clinical outcomes have a higher prevalence of comorbidities, such as chronic obstructive pulmonary disease, diabetes, hypertension, and malignancy 10. It is important to evaluate the risk of adverse outcomes in COVID-19 patients by stratified analysis for comorbidities. However, it is difficult to integrate all of the comorbidity information simultaneously when evaluating clinical outcome. Therefore, several measures have been designed to evaluate the overall impact of comorbidities, including the Charlson (CCI) 11 and Elixhauser (ECI) comorbidity indices 12. To our knowledge, no prognostic model based on comorbidities and clinical information has been reported for COVID-19 patients.

Most mild patients have good prognoses, although the outcomes of critically ill patients are unclear. Therefore, this study established prognostic scoring models based on comorbidities and clinical information, which may aid in evaluating the outcomes of and formulating medical strategies for severe and critical COVID-19 patients.

## Material and Methods

### Study participants

The patient records used in this study were obtained from the First Affiliated Hospital of Wenzhou Medical University (Wenzhou, Zhejiang, China) after ethics committee approval. The requirement for individual patient consent was waived for this study because it did not affect clinical care and all health information was deidentified.

Patients diagnosed with COVID-19 using the criteria of the Diagnosis and Treatment of Novel Coronavirus Pneumonia of China 13 (trial version 5) who received medical treatment for severe illness at the First Affiliated Hospital of Wenzhou Medical University were enrolled. Fifty-one patients were admitted between January 29, 2020, and February 18, 2020. In addition to the epidemiological history and clinical features, all patients included in our study were confirmed by positive SARS-CoV-2 nucleic acids using real-time reverse-transcription polymerase chain reaction detection of throat swabs or lower respiratory tract specimens. According to the clinical classification of the National Health Committee of China 13, the COVID-19 patients were divided into four types: mild, typical, severe, and critical. The mild and typical cases were excluded from this study.

Severe illness met at least one of the following criteria: (1) respiratory rate ≥ 30/minute, (2) finger oxygen saturation ≤ 93% at rest, and (3) arterial partial pressure of oxygen/inspiratory oxygen fraction ≤ 300 mmHg 13. Critical illness met at least one of the following criteria: (1) respiratory failure with mechanical ventilation, (2) shock, and (3) transferred to the intensive care unit due to multiple organ failure 13.

### Data collection

The data extracted from the electronic medical records of the First Affiliated Hospital of Wenzhou Medical University comprised gender, age, smoking history, comorbidities, initial symptoms, respiratory therapy strategies, medications, laboratory data, and hospital length of stay (LOS). The baseline laboratory data were measured during the first 24 hours of admission. Clinical outcomes were followed up to March 16, 2020. Patients were allowed to leave the hospital only when they met the discharge standards of the National Health Committee of China 13.

### Comorbidity assessment

Information on individual comorbidities before the diagnosis of COVID-19 was obtained via oral reports by the patients and their families. Comorbidities were assessed using the following four indexes: CCI, ECI, age- and smoking-adjusted Charlson comorbidity index (ASCCI), and age- and smoking-adjusted Elixhauser comorbidity index (ASECI). The CCI 11 and ECI 12, 14 are comorbidity scoring systems for 17 and 30 different medical conditions, respectively. **Tables S1** and** S2** give the details of the comorbidities the CCI and ECI are based on. The ASCCI and ASECI were built from the CCI and ECI after adding points for age and smoking.

### Outcome variables

The primary outcome was hospital LOS, divided into short (≤ 24 days) and long (> 24 days) LOS. The secondary outcome was progression to critical illness.

### Statistical analysis

Kolmogorov-Smirnov normality tests were used to evaluate the normality assumption for numerical variables. Normally distributed data were expressed as the mean ± standard deviation and non-normally distributed data as the median and inter-quartile range. Categorical variables were presented as a frequency with a percentage. Inter-group differences for the normally and non-normally distributed variables were compared using the unpaired Student's* t*-test and Wilcoxon rank-sum test, respectively. The Pearson χ^2^ test and Fisher's exact test were used to analyze categorical variables.

To assess the association of clinical variables with hospital LOS in the COVID-19 patients, we first screened gender, age, smoking history, white blood cell (WBC), lymphocyte, d-dimer, and interleukin-6 (IL-6), which are reported to be related to prognosis. To avoid over-fitting, we removed the variables that were not associated with the outcome via univariate analysis (*P*-value ≥ 0.1). Then, the significant variables (*P*-value < 0.1, *i*.*e*., former/current smoking and age) in the multivariate model were assigned the corresponding scores matching the CCI and ECI according to the regression coefficient. This gave two new scoring models (ASCCI and ASECI) for predicting outcomes.

The ability of the four models (CCI, ECI, ASCCI, and ASECI) to predict prognosis was examined by logistic regression. The results are presented as odds ratios (ORs) with 95% confidence intervals (CIs). Model discrimination was also assessed by calculating the receiver operating characteristic (ROC) curves and area under the receiver operating characteristic curve (AUROC). The sensitivity and specificity of the models were determined by ROC curve analysis. The DeLong test was used to evaluate differences in AUROC among the models.

A two-sided P-value < 0.05 was regarded as representing statistical significance. Additionally, we used a wider P-value (< 0.1) to filter potentially relevant variables in the univariate analysis. Statistical analyses were performed using SPSS software 20.0 (SPSS, Chicago, IL, USA) and MedCalc software 19.0.5 (MedCalc Software, Ostend, Belgium).

## Results

### Clinical characteristics

The clinical characteristics of 51 patients with COVID-19 were summarized in **Table 1**. There were significant differences in scores of CCI, ECI, ASCCI and ASECI between short-term and long-term LOS group. One patient was classified in long-term LOS group even though she died 14 days after admission. The mean LOS of the COVID-19 patients was 22.82 ± 12.32 days, and 19 patients (37.3%) were > 24 days. The detailed distribution of hospital LOS was presented in **Figure S1**.

### Analysis of clinical variables

We analysed the relationships between 7 clinical variables (gender, age, smoking history, WBC, lymphocyte, d-dimer and IL-6) and hospital LOS. As shown in **Table S3**, statistical differences between age (OR = 1.065, P = 0.012, 95%CI = 1.014-1.119), smoking history (OR = 4.083, P = 0.049, 95%CI = 1.005-16.597), IL-6 (OR = 1.011, P = 0.080, 95%CI = 0.999-1.022) and hospital LOS was identified via univariate analysis. Then, IL-6 was removed from the final multivariable model (forward LR method) because P-value was ≥ 0.1. The final independent variables of the multivariable model were presented in **Table 2**. Lastly, the corresponding scores were assigned to former/current smoking group and different age groups according to regression coefficient.

### Four models of prognostic evaluation based on comorbidities

The detailed distribution of comorbidities based on the CCI and ECI was presented in **Tables S1 and S2**. In the CCI model, a total of 27 patients (52.9%) had no comorbidity, and the most common comorbid condition was mild liver disease (11 patients, 21.6%). As to the ECI model, a total of 16 patients (31.4%) had no comorbidity, and the most common comorbid condition was hypertension (19 patients, 37.3%).

As shown in **Table 3**, there were significant trends for increasing hospital LOS with increasing scores of CCI, ASCCI and ASECI (OR = 57.500, P = 0.001, 95%CI = 5.687-581.399, OR = 71.500, P = 0.001, 95%CI = 5.689-898.642, OR = 19.556, P = 0.001, 95%CI = 3.315-115.372, respectively). A similar result can be drawn from the outcome of critical illness (OR = 21.333, P = 0.001, 95%CI = 3.565-127.672, OR = 13.000, P = 0.009, 95%CI = 1.921-87.990, OR = 11.333, P = 0.008, 95%CI = 1.859-69.080, respectively).

**Figure 1:**
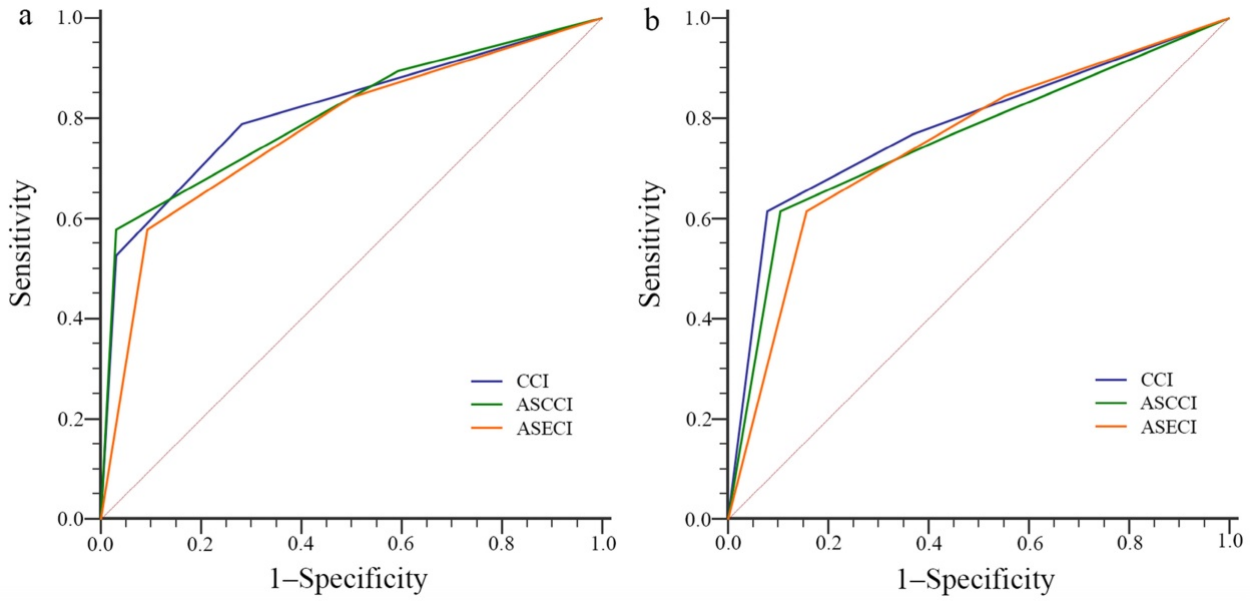
ROC curve of the different models for the outcomes of (a) hospital LOS and (b) critical illness. ASCCI, age and smoking-adjusted Charlson comorbidity index; ASECI, age and smoking-adjusted Elixhauser comorbidity index; CCI, Charlson comorbidity index; LOS, length of stay; ROC, receiver operating characteristic.

Comparisons of the performance among the different models, based on the sensitivity, specificity, ROC curves and AUROC, were summarized in **Table 4** and **Figure 1a, b**. All the three models showed good performance, however, there were numerical differences but no statistical differences in the AUROC values among the three models via DeLong test.

## Discussion

This study found significant associations of the hospital LOS with clinical characteristics, including age, smoking history, IL-6, and comorbidities. Furthermore, we developed prognostic scoring models based on existing comorbidity indices to evaluate the outcomes of severe and critical COVID-19. The CCI, ASCCI, and ASECI models performed well and helped clinical-decision making.

In just a few months, the number of confirmed cases of COVID-19 and deaths worldwide has risen rapidly 4. Although the overall mortality rate is lower than those of SARS-CoV and Middle East respiratory syndrome coronavirus 15, severe and critical COVID-19 patients still have poor outcomes and high mortality 15, 16. It is important to identify effective indicators or scoring models that predict their outcomes.

Several studies have confirmed that older age and smoking status are associated with negative progression and poor outcomes of COVID-19 7-9, which was consistent with our findings. Moreover, Li *et al*. found that males were likely to have more complicated clinical conditions and worse in-hospital outcomes than females 17. In addition to demographic characteristics, several laboratory indicators have been reported to be closely related to the prognosis of COVID-19. Gao *et al*. reported that IL-6 and d-dimer were closely related to the occurrence of severe COVID-19 in adults, and their combined detection had the highest specificity and sensitivity for early prediction of the severity of COVID-19 18. Zhou *et al*. believed that d-dimer > 1 μg/mL could help clinicians to identify patients with poor prognosis at an early stage 8. Qu *et al*. showed that the platelet-to-lymphocyte ratio of patients reflected the degree of cytokine storm, and might be a new predictor of the prognosis of COVID-19 19. However, no significant correlations of hospital LOS with gender, IL-6 and d-dimer were found in our study.

Examining the impact of comorbidities on the outcome of COVID-19, population analyses of the COVID-19 patients with cancer 20 and diabetes 21 found that the patients with either were more likely to have rapid progression and poor outcomes. Furthermore, Guan *et al*. evaluated the risk of a serious adverse outcome in patients with COVID-19 by stratification according to the number and type of comorbidities, identifying sub-populations with poorer prognoses 10. However, no scoring system integrating all comorbidities has been established for evaluating clinical outcomes in COVID-19. Therefore, we evaluated a system for scoring comorbidities to evaluate their impact on the prognosis of severe and critical COVID-19, comparing the CCI, ECI, ASCCI, and ASECI comorbidity models to determine which one is the best outcome predictor.

The CCI includes 17 comorbidities and was first developed to predict 1-year mortality using data for one hospital and was validated in a cohort of 685 breast cancer patients from another hospital 11. The CCI is the most widely used comorbidity index and has long proven useful 22-24. Modification of the CCI, after adjusting for other significant covariates such as age could improve the predictive ability of the model 25. The ECI includes 30 comorbid conditions and is used to predict in-hospital mortality 12. Simard *et al*. established a new index combining the CCI and ECI that could predict the 30-day mortality in the general population 26. However, the application of CCI and ECI to acute infectious diseases is still in its infancy. Therefore, this paper is an exploratory study.

Our study has several limitations. First, it was a single-center, retrospective study with a small sample size, so confounding factors and selection bias are inevitable. Second, the generalization ability of the models was not validated externally. Further studies need to validate these models using new data from different medical centers. Third, the original weights of CCI and ECI were derived using inpatient data from a hospital and they were not COVID-19-specific. Therefore, it is necessary to construct COVID-19-specific weights for a future comorbidity scoring model. Fourth, under-reporting of comorbidities should not be ignored as a major limitation, as it may lead to biased results. However, significant under-reporting was unlikely because our findings were largely consistent with previous studies 5, 6, 10.

## Conclusions

Older age, smoking, and a high comorbidity score were most likely to be associated with poor prognoses for severe and critical COVID-19 cases. We established prognostic scoring models based on comorbidities and clinical information that might help the graded management of patients with different prognosis scores and remind physicians to pay more attention to patients with high risk scores.

## Supplementary Material

Supplementary figure and tables.

## Figures and Tables

**Table 1:** Clinical characteristics of the severe and critical patients with COVID-19

Characteristics	Total	Short-term LOS (≤ 24 days)	Long-term LOS (> 24 days)
	(n = 51)	(n = 32)	(n = 19)
Gender (men/women)	36/15	22/10	14/5
Age (years)	57.37 ± 14.98	53.09 ± 13.63	64.58 ± 14.69^**^
< 40, n (%)	3 (5.9)	3 (9.4)	0 (0)
≥ 40, < 50, n (%)	12 (23.5)	10 (31.3)	2 (10.5)
≥ 50, < 60, n (%)	14 (27.5)	7 (21.9)	7 (36.8)
≥ 60, < 70, n (%)	10 (19.6)	9 (28.1)	1 (5.3)
≥ 70, < 80, n (%)	9 (17.6)	3 (9.4)	6 (31.6)
≥ 80, < 90, n (%)	2 (3.9)	0 (0)	2 (10.5)
≥ 90, n (%)	1 (2.0)	0 (0)	1 (5.3)
Smoking history			
Former/current, n (%)	40 (78.4)	28 (87.5)	12 (63.2)
Never, n (%)	11 (21.6)	4 (12.5)	7 (36.8)
Initial symptoms			
Fever, n (%)	48 (94.1)	30 (93.8)	18 (94.7)
Chill, n (%)	16 (31.4)	14 (43.8)	2 (10.5)^*^
Pharyngodynia, n (%)	5 (9.8)	2 (6.2)	3 (15.8)
Cough, n (%)	38 (74.5)	22 (68.8)	16 (84.2)
Sputum, n (%)	22 (43.1)	12 (37.5)	10 (52.6)
Fatigue, n (%)	7 (13.7)	6 (18.8)	1 (5.3)
Headache/dizziness, n (%)	2 (3.9)	2 (6.2)	0 (0)
Myalgia, n (%)	5 (9.8)	4 (12.5)	1 (5.3)
Conjunctival congestion, n (%)	0 (0)	0 (0)	0 (0)
Diarrhea, n (%)	2 (3.9)	2 (6.2)	0 (0)
Chest distress/dyspnea, n (%)	10 (19.6)	5 (15.6)	5 (26.3)
Respiratory therapies			
High-flow oxygen therapy, n (%)	21 (41.2)	5 (15.6)	16 (84.2)^**^
Noninvasive ventilation, n (%)	15 (29.4)	2 (6.2)	13(68.4)^**^
Invasive ventilation, n (%)	9 (17.6)	0 (0)	9 (47.4)^**^
ECMO, n (%)	6 (11.8)	0 (0)	6 (31.6)^**^
Medication			
Antiviral therapy, n (%)	51 (100)	32 (100)	19 (100)
Antibiotic therapy, n (%)	46 (90.2)	27 (84.4)	19 (100)
Glucocorticoid therapy, n (%)	27 (52.9)	13 (40.6)	14 (73.7)^*^
Intravenous immunoglobulin therapy, n (%)	21 (41.2)	12 (37.5)	9 (47.4)
Thymosin therapy, n (%)	24 (47.1)	11 (34.4)	13 (68.4)^*^
Laboratory data			
WBC (10^9^/L)	7.78 (5.31-11.32)	6.78 (4.84-10.88)	9.95 (7.44-12.36)^*^
Lymphocyte (10^9^/L)	0.81 (0.54-1.11)	0.81 (0.54-1.01)	0.81 (0.48-1.21)
D-dimer (mg/L)	0.85 (0.60-1.24)	0.84 (0.55-1.17)	0.91 (0.68-1.58)
IL-6 (pg/mL)	12.71 (4.04-74.65)	8.35 (3.88-47.84)	34.00 (4.42-103.34)
CCI	0.00 (0.00-1.00)	0.00 (0.00-1.00)	2.00 (1.00-3.00)^**^
ECI	0.00 (0.00-9.00)	0.00 (0.00-0.00)	9.00 (0.00-13.00)^**^
ASCCI	2.29 ± 1.78	1.42 ± 0.88	3.76 ± 1.95^**^
ASECI	2.00 (0.50-11.00)	1.25 (0.50-2.00)	12.00 (2.50-15.00)^**^
Critical cases, n (%)	13 (25.5)	1 (3.1)	12 (63.2)^**^

Data were expressed as mean ± standard deviation, median (inter-quartile range) or frequency (percentage). ^*^, P < 0.05; ^**^, P < 0.01. ASCCI, age and smoking-adjusted Charlson comorbidity index; ASECI, age and smoking-adjusted Elixhauser comorbidity index; CCI, Charlson comorbidity index; COVID-19, Corona Virus Disease 2019; ECI, Elixhauser comorbidity index; ECMO, extracorporeal membrane oxygenation; IL-6, interleukin-6; LOS, length of stay; WBC, white blood cell.

**Table 2:** The final parameters of the multivariable model

Independent variables	Points	Regression coefficient	OR	P-value	95%CI
Age (years)		0.62	1.064	0.018	1.011-1.121
< 40	0				
≥ 40, < 50	0.5				
≥ 50, < 60	1.0				
≥ 60, < 70	1.5				
≥ 70, < 80	2				
≥ 80, < 90	2.5				
≥ 90	3				
Smoking history		1.307	3.696	0.080	0.856-15.955
Never	0				
Former/current	1				
Constant	-	-4.539	-	0.006	-

The significant P-value was indicated in bold. CI, confidence interval; OR, odds ratio.

**Table 3:** Four models of prognostic evaluation based on comorbidities for the severe and critical patients with COVID-19

Models	LOS (≤ 24 days or > 24 days)			Critical illness (yes or no)		
	OR	P-value	95%CI	OR	P-value	95%CI
CCI model						
= 0 (n = 27)	Reference			Reference		
= 1 (n = 13)	3.594	0.104	0.769-16.787	1.455	0.703	0.212-9.984
≥ 2 (n = 11)	57.500	**0.001**	5.687-581.399	21.333	**0.001**	3.565-127.672
ECI model						
< 0 (n = 5)	Reference			Reference		
= 0 (n = 28)	0.250	0.191	0.031-1.999	0.480	0.565	0.040-5.831
> 0 (n = 18)	3.900	0.196	0.494-30.758	4.000	0.253	0.371-43.139
ASCCI model						
≤ 1 (n = 15)	Reference			Reference		
> 1, ≤ 3 (n = 24)	2.167	0.387	0.376-12.495	0.929	0.940	0.136-6.323
> 3 (n = 12)	71.500	**0.001**	5.689-898.642	13.000	**0.009**	1.921-87.990
ASECI model						
≤ 1 (n = 19)	Reference			Reference		
> 1, ≤ 5 (n = 18)	2.051	0.381	0.411-10.238	1.700	0.588	0.249-11.586
> 5 (n = 14)	19.556	**0.001**	3.315-115.372	11.333	**0.008**	1.859-69.080

The significant P-value was indicated in bold. ASCCI, age and smoking-adjusted Charlson comorbidity index; ASECI, age and smoking-adjusted Elixhauser comorbidity index; CCI, Charlson comorbidity index; CI, confidence interval; COVID-19, Corona Virus Disease 2019; ECI, Elixhauser comorbidity index; LOS, length of stay; OR, odds ratio.

**Table 4:** Performance comparisons among the different models

Models	Sensitivity	Specificity	AUROC	SE	95%CI	P-value
For LOS						
CCI model	79.0	71.9	0.816	0.063	0.682-0.910	< 0.0001
ASCCI model	57.9	96.9	0.808	0.063	0.674-0.905	< 0.0001
ASECI model	57.9	90.6	0.776	0.068	0.638-0.881	< 0.0001
For critical illness						
CCI model	61.5	92.1	0.783	0.081	0.646-0.886	0.0005
ASCCI model	61.5	89.5	0.758	0.085	0.618-0.867	0.0023
ASECI model	61.5	84.2	0.750	0.080	0.609-0.861	0.0019

The values of sensitivity and specificity were expressed as percentage (%). ASCCI, age and smoking-adjusted Charlson comorbidity index; ASECI, age and smoking-adjusted Elixhauser comorbidity index; AUROC, area under receiver operating characteristic curve; CCI, Charlson comorbidity index; CI, confidence interval; LOS, length of stay; SE, standard error.

## Abbreviations

ASCCI: age and smoking-adjusted Charlson comorbidity index
ASECI: age and smoking-adjusted Elixhauser comorbidity index
AUROC: area under the receiver operating characteristic curve
CCI: Charlson comorbidity index
CI: confidence intervals
COVID-19: Corona Virus Disease 2019
ECI: Elixhauser comorbidity index
IL-6: interleukin-6
LOS: length of stay
OR: odds ratios
ROC: receiver operating characteristic
SARS-CoV: severe acute respiratory syndrome coronavirus
WBC: white blood cell
WHO: World Health Organization
