# Supplementary material for: Prognosis models for severe and critical COVID-19 based on the Charlson and Elixhauser comorbidity indices
Source: Int J Med Sci. 2020 Aug 25;17(15):2257–63. doi: 10.7150/ijms.50007 (PMC7484649; doi:10.7150/ijms.50007)
Supplement: Supplementary file 1 — Supplementary figure and tables. [file ijmsv17p2257s1.pdf]

Table S1 Distribution of Charlson comorbidities in the severe and critical patients with COVID-19

Comorbidities	Total (n = 51)	Short-term LOS (≤ 24 days) (n = 32)	Long-term LOS (> 24 days) (n = 19)	Points
Myocardial infarction, n (%)	1 (2.0)	0 (0)	1 (5.3)	1
Congestive heart failure, n (%)	2 (3.9)	0 (0)	2 (10.5)	1
Peripheral vascular disease, n (%)	10 (19.6)	2 (6.2)	8 (42.1)**	1
Cerebrovascular disease, n (%)	2 (3.9)	0 (0)	2 (10.5)	1
Dementia, n (%)	1 (2.0)	0 (0)	1 (5.3)	1
Chronic pulmonary disease, n (%)	1 (2.0)	0 (0)	1 (5.3)	1
Rheumatic disease, n (%)	0 (0)	0 (0)	0 (0)	1
Peptic ulcer disease, n (%)	0 (0)	0 (0)	0 (0)	1
Mild liver disease, n (%)	11 (21.6)	3 (9.4)	8 (42.1)*	1
Diabetes mellitus without end-organ damage, n (%)	7 (13.7)	5 (15.6)	2 (10.5)	1
Diabetes mellitus with end-organ damage, n (%)	3 (5.9)	0 (0)	3 (15.8)	2
Hemiplegia, n (%)	0 (0)	0 (0)	0 (0)	2
Renal disease, n (%)	0 (0)	0 (0)	0 (0)	2
Any malignancy, including lymphoma and leukemia, except malignant neoplasm of skin, n (%)	2 (3.9)	0 (0)	2 (10.5)	2
Moderate liver disease, n (%)	0 (0)	0 (0)	0 (0)	3
Metastatic solid tumor, n (%)	0 (0)	0 (0)	0 (0)	6
AIDS/HIV, n (%)	0 (0)	0 (0)	0 (0)	6

*, $P < 0.05$; **, $P < 0.01$. AIDS, acquired immune deficiency syndrome; COVID-19, Corona Virus Disease 2019; HIV, human immunodeficiency virus; LOS, length of stay.

Table S2 Distribution of Elixhauser comorbidities in the severe and critical patients with COVID-19

Comorbidities	Total (n = 51)	Short-term LOS (≤ 24 days) (n = 32)	Long-term LOS (> 24 days) (n = 19)	Points
Congestive heart failure, n (%)	2 (3.9)	0 (0)	2 (10.5)	7
Cardiac arrhythmias, n (%)	1 (2.0)	0 (0)	1 (5.3)	5
Valvular disease, n (%)	3 (5.9)	0 (0)	3 (15.8)	–1
Pulmonary circulation disorders, n (%)	1 (2.0)	0 (0)	1 (5.3)	4
Peripheral vascular disorders, n (%)	10 (19.6)	2 (6.2)	8 (42.1)**	2
Hypertension, n (%)	19 (37.3)	10 (31.2)	9 (47.4)	0
Paralysis, n (%)	1 (2.0)	0 (0)	1 (5.3)	7
Neurodegenerative disorders, n (%)	0 (0)	0 (0)	0 (0)	6
Chronic pulmonary disease, n (%)	1 (2.0)	0 (0)	1 (5.3)	3
Diabetes, uncomplicated, n (%)	7 (13.7)	5 (15.6)	2 (10.5)	0
Diabetes, complicated, n (%)	3 (5.9)	0 (0)	3 (15.8)	0
Hypothyroidism, n (%)	1 (2.0)	1 (3.1)	0 (0)	0
Renal failure, n (%)	0 (0)	0 (0)	0 (0)	5
Liver disease, n (%)	11 (21.6)	3 (9.4)	8 (42.1)*	11
Peptic ulcer disease excluding bleeding, n (%)	0 (0)	0 (0)	0 (0)	0
AIDS/HIV, n (%)	0 (0)	0 (0)	0 (0)	0
Lymphoma, n (%)	1 (2.0)	0 (0)	1 (5.3)	9
Metastatic cancer, n (%)	0 (0)	0 (0)	0 (0)	12
Solid tumor without metastasis, n (%)	1 (2.0)	0 (0)	1 (5.3)	4
Rheumatoid arthritis/collagen vascular diseases, n (%)	0 (0)	0 (0)	0 (0)	0
Coagulopathy, n (%)	0 (0)	0 (0)	0 (0)	3
Obesity, n (%)	3 (5.9)	2 (6.2)	1 (5.3)	–4

Weight loss, n (%)	0 (0)	0 (0)	0 (0)	6
Fluid and electrolyte disorders, n (%)	1 (2.0)	0 (0)	1 (5.3)	5
Blood loss anemia, n (%)	0 (0)	0 (0)	0 (0)	–2
Deficiency anemia, n (%)	0 (0)	0 (0)	0 (0)	–2
Alcohol abuse, n (%)	6 (11.8)	4 (12.5)	2 (10.5)	0
Drug abuse, n (%)	1 (2.0)	0 (0)	1 (5.3)	–7
Psychoses, n (%)	1 (2.0)	1 (3.1)	0 (0)	0
Depression, n (%)	2 (3.9)	1 (3.1)	1 (5.3)	–3

*, P < 0.05; **, P < 0.01. AIDS, acquired immune deficiency syndrome; COVID-19, Corona Virus Disease 2019; HIV, human immunodeficiency virus; LOS, length of stay.

Table S3 Univariate analysis of clinical variables with hospital LOS

**Variables**	**OR**	**P-value**	**95%CI**
Gender	0.786	0.709	0.222–2.785
Age (years)	1.065	**0.012**	1.014–1.119
Smoking history	4.083	**0.049**	1.005–16.597
WBC (10^9/L)	1.139	0.118	0.968–1.340
Lymphocyte (10^9/L)	1.387	0.694	0.271–7.092
D-dimer (mg/L)	1.397	0.339	0.704–2.772
IL-6 (pg/mL)	1.011	**0.080**	0.999–1.022

The significant P-value was indicated in bold. CI, confidence interval; IL-6, interleukin-6; LOS, length of stay; OR, odds ratio; WBC, white blood cell.

Figure S1

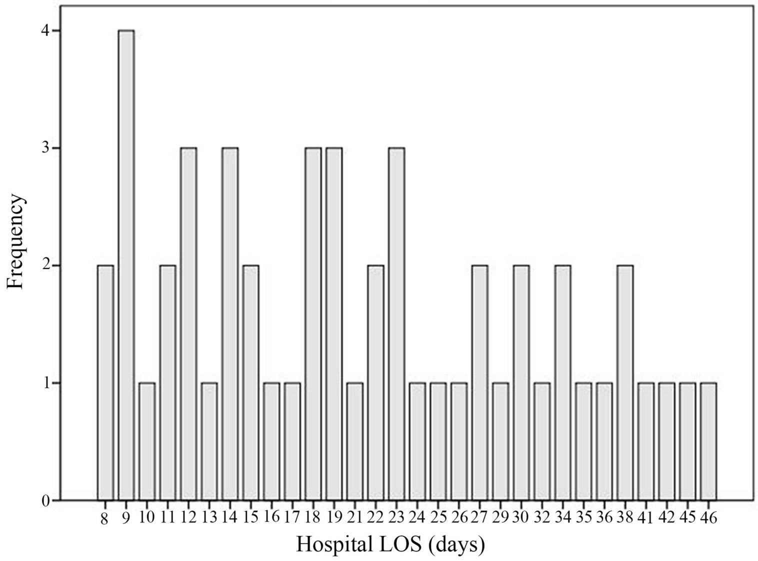

Frequency
4
3
2
1
0
8 9 10 11 12 13 14 15 16 17 18 19 21 22 23 24 25 26 27 29 30 32 34 35 36 38 41 42 45 46
Hospital LOS (days)
